# Mutation of a serine near the catalytic site of the *choline acetyltransferase a* gene almost completely abolishes motility of the zebrafish embryo

**DOI:** 10.1371/journal.pone.0207747

**Published:** 2018-11-20

**Authors:** Swarnima Joshi, Sanamjeet Virdi, Christelle Etard, Robert Geisler, Uwe Strähle

**Affiliations:** Karlsruhe Institute of Technology (KIT), Institute of Toxicology and Genetics (ITG), Eggenstein-Leopoldshafen, Germany; Weizmann Institute of Science, ISRAEL

## Abstract

In zebrafish, the gene *choline acetyltransferase a* (*chata*) encodes one of the two ChAT orthologs responsible for the synthesis of acetylcholine. Acetylcholine (ACh) is essential for neuromuscular transmission and its impaired synthesis by ChAT can lead to neuromuscular junction disorders such as congenital myasthenic syndromes in humans. We have identified a novel mutation in the *chata* gene of zebrafish, *chata*^*tk64*^, in a collection of uncharacterised ENU-induced mutants. This mutant carries a missense mutation in the codon of a highly conserved serine changing it to an arginine (S102R). This serine is conserved among ChATs from zebrafish, rat, mice and chicken to humans. It resides within the catalytic domain and in the vicinity of the active site of the enzyme. However, it has not been reported so far to be required for enzymatic activity. Modelling of the S102R variant change in the ChAT protein crystal structure suggests that the change affects protein structure and has a direct impact on the catalytic domain of the protein which abolishes embryo motility almost completely.

## Introduction

Choline acetyltransferase (ChAT) is responsible for the synthesis of the neurotransmitter acetylcholine (ACh), characteristic for the cholinergic neurons of the peripheral and central nervous system (CNS). ChAT catalyses the reversible synthesis of ACh from acetyl-CoA and choline [1, 2]. In the peripheral nervous system, ACh stimulates muscle contraction and in the central nervous system it facilitates learning as well as short-term memory formation [2].

In humans, abnormal ChAT activity and impaired ACh synthesis are linked to a number of neurodegenerative disorders, including Alzheimer disease, Huntington’s disease, schizophrenia, sudden infant death syndrome and amyotrophic lateral sclerosis [3]. Several recessive mutations have been identified in human ChAT which cause congenital myasthenic syndrome associated with episodic apnea (CMS-EA) [4–6]. The disorder results in severe muscular weakness and respiratory insufficiency with varied severity among patients. It has been shown that CMS-EA caused by mutations in human ChAT is not due to abnormal ACh release in the axon terminals, but to the impaired re-synthesis of ACh after uptake of recycled choline in the axons [6,7].

Studies utilizing site directed mutagenesis in cDNA clones of Drosophila and rat ChAT have shown that highly conserved histidine and arginine are critical for the enzymatic activity [8–10]. Mutagenesis studies in rat ChAT have further shown that arginine 452 interacts with acetyl-CoA, and mutation of this arginine in human ChAT leads to reduced affinity between substrate and enzyme [10]. Histidine 324 in human ChAT (PDB ID 2fy2) [11] is essential for the catalysis [2] and acts as a general site for nucleophilic attack on choline or acetyl-CoA depending on the reaction’s direction [8,12]. A number of studies have focused on the biochemical impact of mutations in ChAT that were identified in patients suffering from congenital myasthenic syndrome with episodic apnea (CMS-EA) [2,7,13]. However, only a small number of such mutations are known in the human population, many of which may be hypomorphic. Thus, the identification of novel amino acid changes that lead to a reduction or loss of ChAT activity can be of great value to understand the function of ChAT.

Here we report a novel missense mutation in a highly conserved serine residue, S102 of the zebrafish gene *choline acetyltransferase a*, causing the embryo to be largely immotile. The embryos do not show an escape response on touch, though some residual motility remains. Sequence homology to human ChAT indicates that the mutation resides within the catalytic domain of the protein near the active site histidine. In silico modelling of protein stability suggests S102R affects ChATa function by rendering it unstable.

## Results

**Fig 1:**
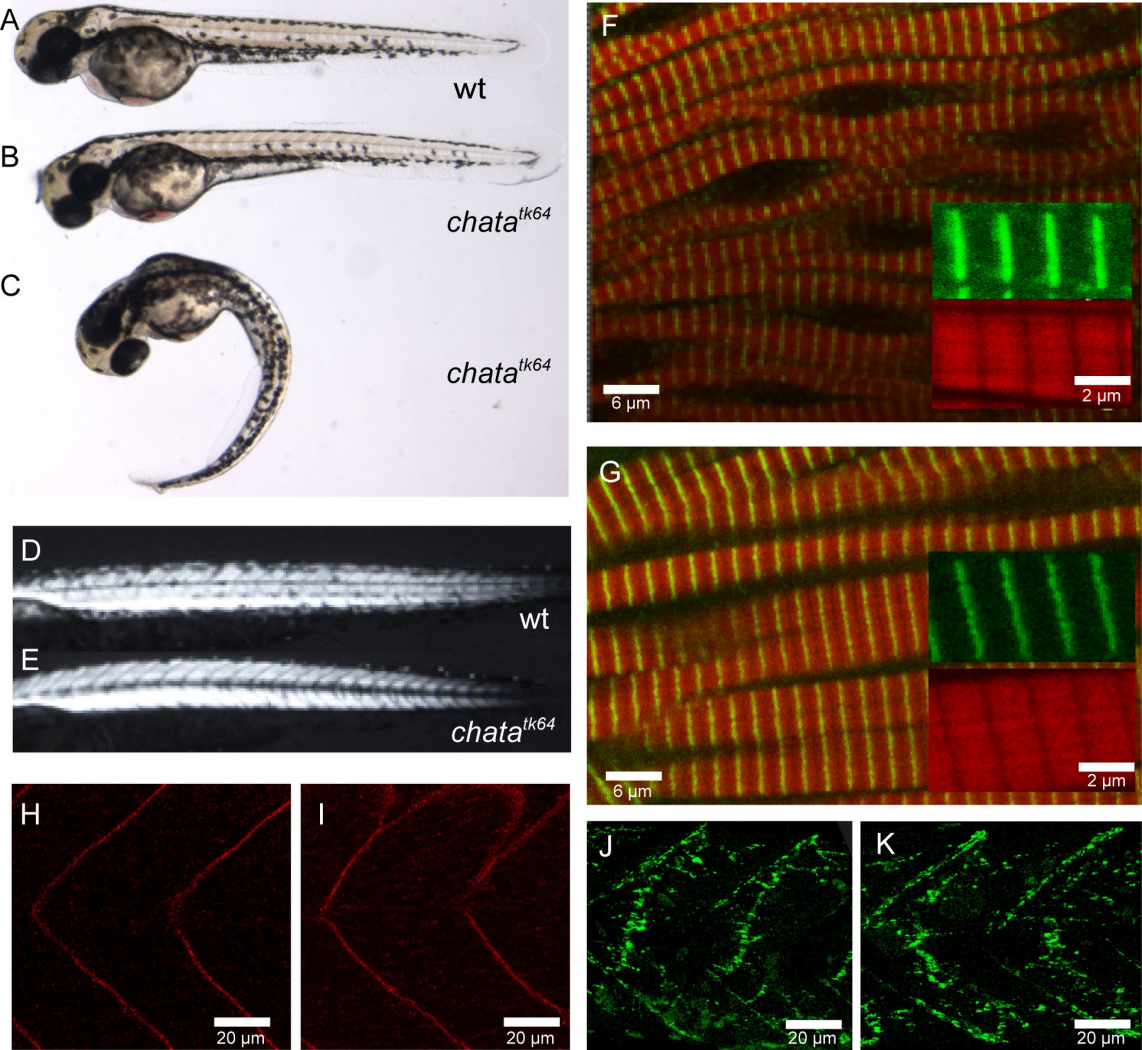
Phenotype of *chata*^*tk64*^. Wild-type embryo **(A)**, *chata*^*tk64*^ mutant 3 hours after de-chorionation **(B)** and immediately after de-chorionation **(C)**. Note that **(B)** is indistinguishable from **(A)**. Birefringence is similar in wild-type **(D)** and *chata*^*tk64*^ mutant **(E)**. Immunohistochemistry with phalloidin (red) marking F-actin, and α-actinin (green) **(F, G)**, β-sarcoglycan **(H, I)** and α-bungarotoxin **(J, K)** do not reveal differences between wild-type **(G, I, K)** and chata^tk64^ mutant **(F, H, J)** at 48 hpf.

The mutant allele *tk64*, exhibiting reduced embryonic motility from 24 hours post-fertilization (hpf) onwards, was identified in a previous large-scale mutagenesis screen for N-ethyl-N-nitrosourea (ENU) induced zebrafish mutants [14] but remained so far uncharacterised. Homozygous *tk64* mutant embryos do not hatch and require manual dechorionation. Initially the body axis of mutants is curved (Fig 1C). Three hours after dechorionation, they straighten up and become indistinguishable from wild-type embryos (Fig 1A and 1B). Touch stimulation results in a considerably reduced response in the mutants compared to that of wild-type embryos: At 48 hpf mutants only twitch in response to touch whereas the wild-type larvae escape from the stimulus, shown in S1 and S2 Videos. The mutant response to touch worsens with time although some residual motility is still present in the mutant at 72 hpf. Mutants exhibit a very small amplitude of tail bending and therefore do not manage to escape the stimulus. The heart beats at normal rate at 48 hpf but decreases over time and ceases around 5 dpf, resulting in death. Furthermore, the swim bladder fails to inflate. Heterozygotes do not exhibit any mobility or other obvious defects and become healthy adults.

### Muscle structure is unaffected in the mutant

Evaluation of muscle integrity by birefringence of polarized light indicated that the overall structure of the myofibrils is not markedly disrupted in 48 hpf embryos (Fig 1D–1E, 48 hpf). Immunostaining confirmed that the myofibrillar components, α-actinin and F-actin are correctly arranged in Z and I bands respectively (Fig 1F–1G) in the mutant, and that titin and slow muscle myosin are correctly localised. The myosepta required for proper anchoring of muscle fibers do not show any disruption as revealed by β-sarcoglycan immunostaining (Fig 1H–1I). We next examined the subcellular distribution of mutant nicotinic acetylcholine receptors (nAChR) by labelling with Alexa Fluor488-conjugated bungarotoxin, which binds to the α-subunit of the nAChR in the postsynaptic membrane [15]. We found that wild-type and *tk64* embryos show a similar punctate staining indicating nAChR clustering at the neuromuscular junctions. (Fig 1J–1K). We also labelled embryos at 72 hpf with an antibody directed against acetylated α-tubulin to visualize the axons of motor neurons. Neither motor axons nor other axonal projections such as those of the Rohon-Beard sensory neurons showed obvious defects.

### The *tk64* allele represents a missense mutant of zebrafish *chata*

**Fig 2:**
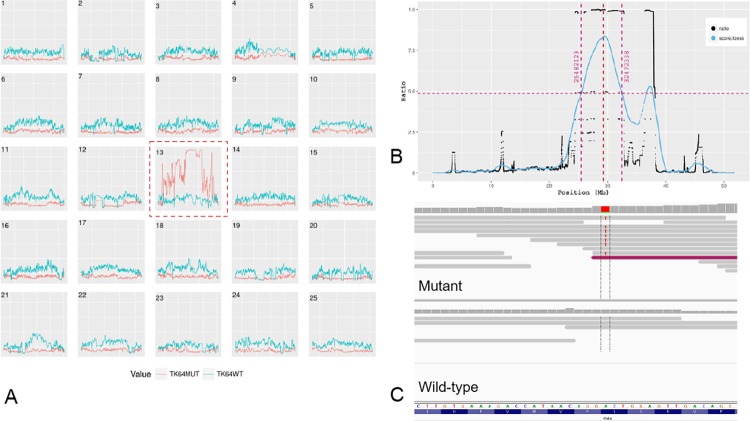
Linkage mapping of *chata*^*tk64*^. **(A)** Plot of all chromosomes showing the homozygosity score in mutant (red) and wild-type (blue) samples, with chromosome 13 showing elevated homozygosity. **(B)** LOESS fit (in blue) of ratio of fraction of homozygosity and fraction of heterozygosity of chromosome 13 yielding a clearly visible region linked to the best candidate mutation in the middle of the peak (red dotted line). **(C)** Integrated Genomics Viewer (IGV) screen-shot of the base change in the mutant with respect to forward strand: Chr13: 29,288,858 A to T.

To determine the underlying mutation resulting in the *tk64* phenotype, we performed linkage mapping by whole genome sequencing and SNP analysis. We identified a region co-segregating with the mutation on chromosome 13 by applying our *in-house* mutation mapping pipeline (manuscript in preparation) (Fig 2A and 2B). A critical region linked to the mutation was identified between 25.48 Mb and 32.47 Mb on chromosome 13 (Fig 2A). All variants inside this region that were homozygous in the mutant sample and heterozygous or homozygous for the reference sequence in the wild-type sample were extracted and annotated (see methods). After filtering out intergenic and low impact variations in coding regions, two missense mutations (Table 1) remained for further analysis: an A to C transversion giving rise to a lysine to glutamine substitution at position 101 in the gene *cxcl18a*.*1* (*chemokine (C-X-C motif) ligand 18a*, *duplicate 1*); and a T to A transversion giving rise to a serine to arginine substitution at position 102 in *chata* (*choline O-acetyltransferase a*) (Fig 2C; Table 1). By applying the SIFT algorithm [16] the mutation in *chata* was predicted to be deleterious (SIFT score of 0) while that in *cxcl18a*.*1* was predicted to be tolerated (SIFT score 3.1). Furthermore, *chata* is known to be involved in locomotion via neuromuscular transmission [17], while *cxcl18a*.*1* is a member of a protein family involved in chemokine activity [18]. For these reasons, *chata* was selected as the most likely candidate gene.

**Table 1:** Candidate mutations in the critical region of *tk64*. Ensembl variant effect predictor (VEP) [19] software identified candidates for missense mutations on chromosome 13 (see Results section for details). A total of 101 variants were identified. Variants with low or "modifier" impact rating [19] were removed leaving variants with moderate impact on only two genes. Transcripts with accession prefix “NM_” are from the RefSeq database. Transcripts with accession prefix “ENSDART” are from the Ensembl database.

Location	Variation	Gene	Transcripts	Consequence	Amino acid	SIFT Score
13:29288858	A to T	*chata*	ENSDART00000024225 ENSDART00000150228 NM_001130719.1 NM_001130719.1	Missense	S to R	deleterious(0)
13:30563448	A to C	*cxcl18a*.*1*	ENSDART00000108949	Missense	K to Q	tolerated(0.31)

It has been previously reported that the zebrafish mutant *bajan* (*chata*^*tf247*^) contains a point mutation at a splice acceptor of zebrafish ChATa which gives rise to 3 different mutated transcripts, all of them introducing a stop codon [17]. Three independent couples generated progeny with close to the expected ratio of 25% of embryos with motility problems similar to those observed in *chata*^*tk64*^ homozygotes (data not shown). Thus, *bajan* and *chata*^*tk64*^ did not complement, indicating that *chata*^*tk64*^ carries a mutation in the zebrafish *chata* gene that leads to a functionally deficient protein.

### Replacement of Serine 102 by arginine in ChATa impairs its function

**Fig 3:**
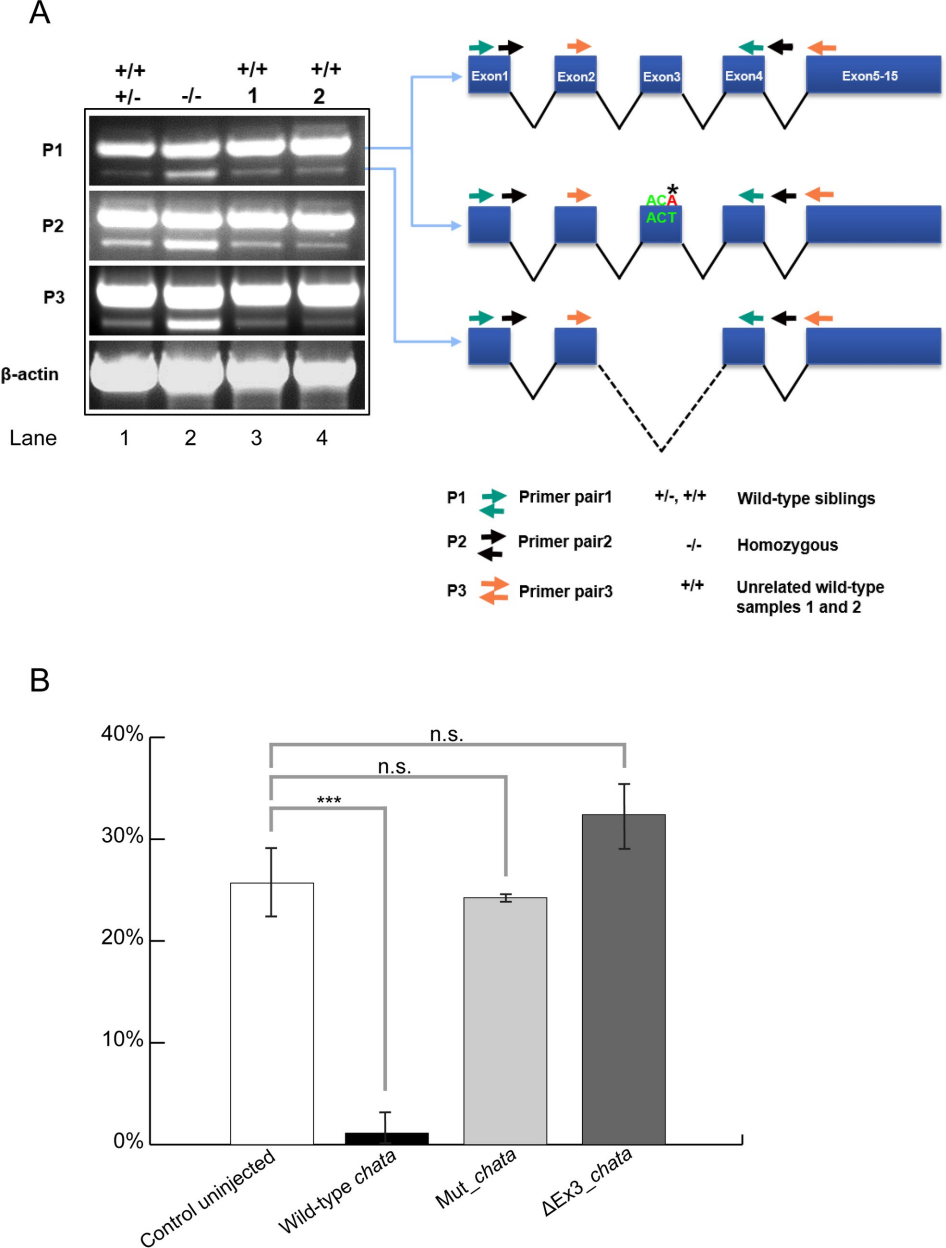
Variants of *chata* mRNA and rescue of the mutant phenotype. A. cDNA fragments amplified by RT-PCR, Left: Overlapping RT-PCR amplicons obtained with three different primer pairs (P1, P2, P3). With each of the primer pair two different PCR products, with and without exon 3 were amplified from wild-type siblings, *chata*^*tk64*^ and unrelated wild-type cDNAs and confirmed by Sanger sequencing. From left, lane 1 wild-type siblings; lane 2: homozygous *chata*^*tk64*^; lane 3 and 4: unrelated wild-type embryos. Right: Schematic diagram of the sequenced PCR products of zebrafish *chata*, top: wild-type; middle: *chata*^*tk64*^; bottom: ΔEx3. For each of the primer pairs, the ΔEx3 product is more pronounced in the mutants than in the siblings or unrelated wild-types. **B.** Wild-type *chata* mRNA rescues the *chata*^*tk64*^ phenotype. Embryos were assessed with respect to their motility phenotype at 72 hpf after injection. In the wild-type mRNA injected embryos only 1% were mutants showing 99% rescue whereas the mRNA with missense mutation as in *chata*^*tk64*^ (Mut_*chata*) and lacking exon 3 (ΔEx3_*chata*) gave a Mendelian ratio of mutants in the injected population. Significance was checked with Student's *t*-test at *P<0*.*05*. Asterisks and "n.s." above the bars represent significant and non-significant *p* values respectively. Data shown is mean ± standard deviation (n ≥ 17).

To further verify that the missense mutation found in *chata*^*tk64*^ is indeed responsible for the observed phenotype, we performed rescue experiments. Wild-type *chata* and the *chata*^*tk64*^ allele were cloned from cDNA derived from wild-type and mutant embryos, respectively (Fig 3A).

Synthetic mRNAs of wild-type and mutant *chata*^*tk64*^ carrying the missense mutation were injected into zygotes obtained from matings of heterozygous *chata*^*tk64*^ parents. Embryos were examined at 48 hpf for their motility by the touch response assay. Since *chata*^*tk64*^ is recessive, 25% homozygous mutant embryos from crosses of heterozygous parents show the mutant phenotype in uninjected controls (Fig 3B). However, only 1% of the embryos resulting from such crosses injected with wild-type *chata* mRNA showed a motility defect upon touch, suggesting behavioural rescue (Fig 3B). The S102R mutant *chata*^*tk64*^ mRNA did not rescue the phenotype (Fig 3B) resulting in approximately 25% motility mutants, thus confirming that the missense mutation causing S102R is responsible for the observed motility phenotype.

In the course of our cDNA amplifications, we noted a shorter fragment that is weakly present in PCR fragments amplified from wild-type and more strongly in PCR fragments from mutant cDNA (Fig 3A). Sequencing of the PCR fragments revealed that these shorter fragments represent the same mRNA variant that lacks exon 3. This suggests that in both wild-type and mutants a *chata* variant is expressed which we refer to ΔEx3. Moreover, its abundance is increased in the *chata*^*tk64*^ mutants. Because exon3 encodes part of the catalytic site of ChAT, the deletion is predicted to abolish its enzymatic activity. To exclude any possibility that this variant contributes to the observed motility phenotype, a synthetic mRNA encoding ΔEx3 was injected into embryos derived from *chata*^*tk64*^ heterozygous parents. This variant was unable to rescue the phenotype: approximately 30% embryos showed the motility defect (Fig 3B). Moreover, the remaining 70% of embryos did not show any additional defects, suggesting that at least under these assay conditions ΔEx3 is non-functional and does not contribute to the observed motility defect phenotype.

### Serine to arginine change in ChATa affects protein secondary structure

**Fig 4:**
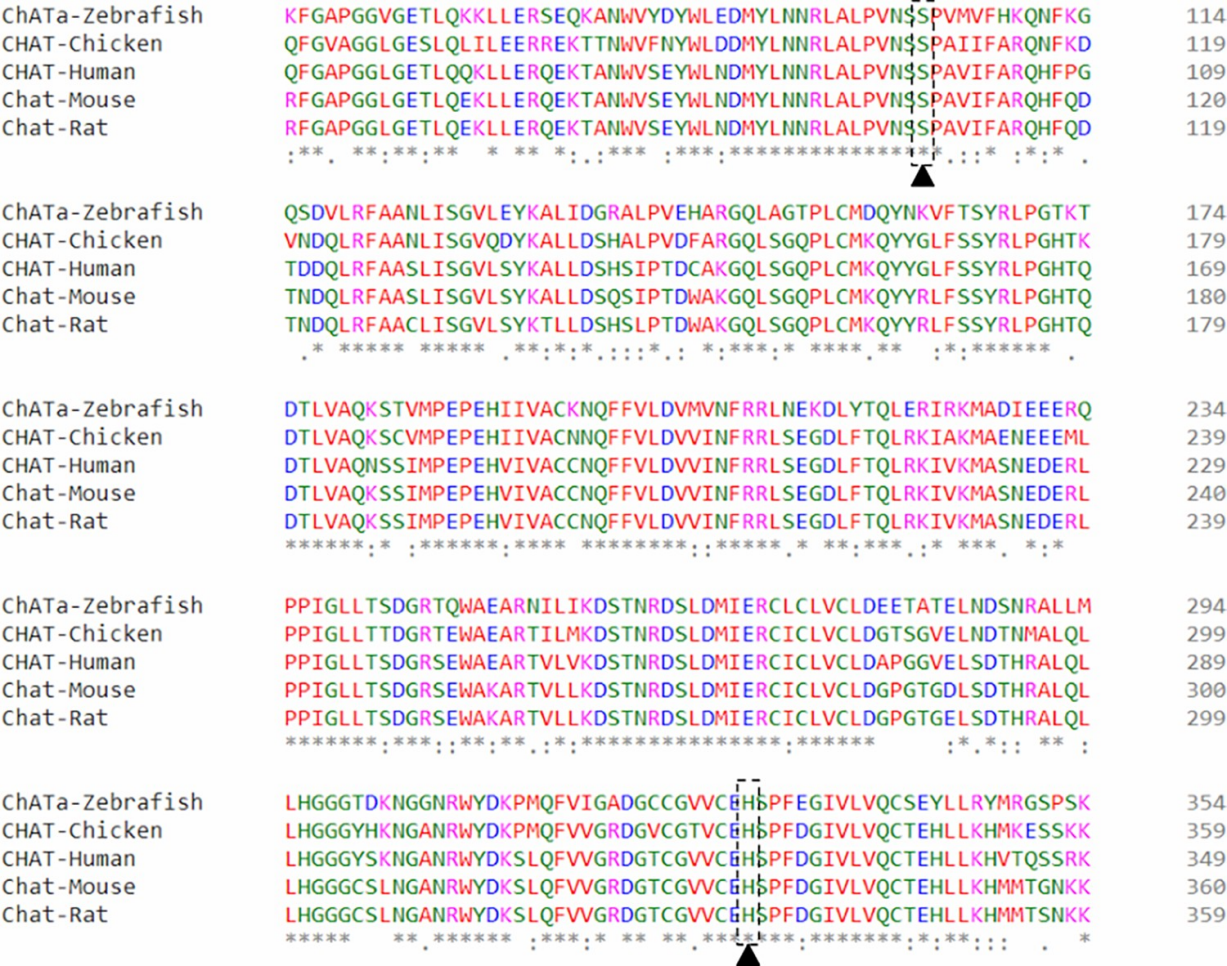
Serine residue 102 of zebrafish ChATa is highly conserved. Multiple sequence alignment (residue range 50–349 in human 70kDa CHAT Isoform R, UniProtKB ID: P28329-3) of vertebrate transcripts showing that serine and histidine (active site) residues are highly conserved among human, mouse, rat and chicken.

**Fig 5:**
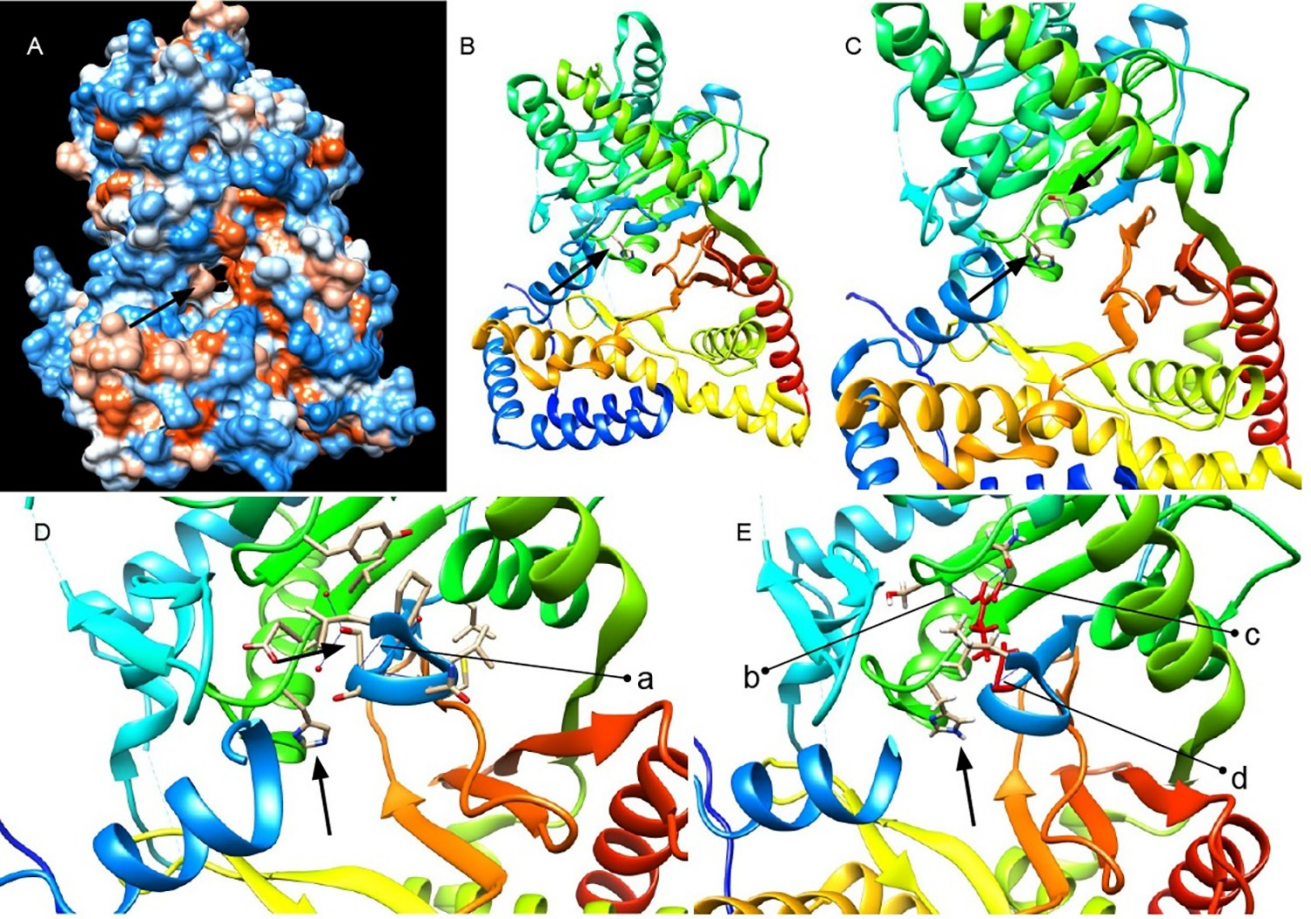
Human ChAT protein crystal structure and predicted effect of the *chata*^*tk64*^ mutation. **(A)** Front view of protein crystal structure of human ChAT showing catalytic tunnel and **(B)** active site histidine H324. Residues 90 to 391 comprise the catalytic domain according to the human ChAT protein structure PDB ID 2fy2 (90–394 in 70 kDa ChAT Isoform R, UniProtKB ID: P28329-3) [11]. The length of the N-terminal domain of the protein differs by 118 residues from the canonical protein isoform M (UniProtKB ID: P28329-1) and the structure starts at residue 8 [7,11]. **(C)** Serine residue in the vicinity of active site in the protein (residues 90–95 are removed from the structure for better visualization). **(D)** Wild-type serine residue S97 forming a salt bridge a = 1.80 Å with residue L92 (residues 88–91 and 235–243 are deleted from the structure for better visualization). **(E)** Serine to arginine mutation S97R after structure minimization in UCSF chimera. R97 (in red) forms a new H-bond with serine S307 b = 2.50 Å; salt bridge with L92 d = 1.80 Å; and two new H-bonds with glutamine 309 c = [2.43 Å and 1.64 Å] (residues 86–90 and 236–246 are deleted from the structure for better visualization).

We investigated the amino acid change in *chata*^*tk64*^ with respect to the protein structure. Zebrafish ChATa S102 is a conserved amino acid among vertebrate orthologues (Fig 4). This corresponds to residue S215 in full length human ChAT isoform 2 (protein sequence: NP065574.1). In the published ChAT protein crystal structure, PDB ID 2fy2, it corresponds to S97 in the 70 kDa isoform of ChAT which is 118 residues shorter at the N-terminus than the 83 kDa isoform [11] (Fig 5C). This residue falls within a range of residues which constitutes a turn/coil between α-helix (residues 92–96) and a β-sheet (residues 99–101). We ran several prediction programs to determine the functional effect of the mutation on the protein structure and function. Using the protein crystal structure of human ChAT (PDB ID 2fy2) [11], and introducing a S97R amino acid change in chain A, the mutation Cutoff Scanning Matrix program (mCSM) [20] predicted an unstable protein. This program measures the difference of the Gibbs free energy of unfolding (in the absence of denaturant) between wild-type and mutant. It resulted in a predicted stability change (ΔΔG) of -0.98 Kcal/mol. This negative value categorizes the change as destabilizing. The functional impact of the S102R substitution was also checked with PANTHER-PSEP (position-specific evolutionary preservation) v9.0 [21] which predicted the change as “probably damaging” because the position in the protein has been evolutionarily conserved for more than 450 million years (Fig 4).

We also performed protein structure prediction for zebrafish ChATa using Phyre^2^ (Protein Homology/analogY Recognition Engine v2.0) [22]. As expected, the structure of the human ChAT [11] scored for the maximum identity of around 68 percent which was then used for ChAT protein structure analysis for the effect of mutation. Serine 97 resides in the catalytic domain of the protein and in the vicinity of active site histidine 324 within a distance of 5 Å (PDB ID 2fy2) (Fig 5A–5C) [11]. Introducing the amino acid change S97R, the protein structure accommodates the arginine without any clashes but the arginine forms new salt bridges and forms hydrogen bonds by its side chain, two with glutamine 309 by its donor NH and NH_2_ groups, and with serine 307 by its donor NH_2_ group (Fig 5E). It maintains the salt bridge with leucine 92 (Fig 5D). Also, arginine replaces the polar uncharged serine and introduces a charge into the buried center of the protein in close vicinity of the active site (Fig 5C and 5E), which possibly affects protein structure and is likely to interfere with the effectiveness of the catalysis which correlates with the predicted SIFT score.

## Discussion

In this study we report a new missense mutation in a highly conserved amino acid which resides in the catalytic domain of ChATa, one of the two zebrafish orthologs of ChAT. *chata*^*tk64*^ shows a strong reduction, but not a complete absence of motility, indicating residual synaptic activity at the neuromuscular junction.

The phenotype resembles that of another mutant *chata* allele, *chata*^*tf247*^ (also called *bajan*), which was previously reported in the zebrafish by Wang et al. [17]. Neither of the mutations has an obvious effect on synaptogenesis. Immunohistochemistry using α-bungarotoxin confirmed no defect at post-synaptic receptors whereas electrophysiological recordings in the *bajan* mutant showed a potential defect in the synaptic transmission: *bajan* shows a decreased quantal size, attributable to incomplete filling of presynaptic vesicles with Ach [17]. The same mechanism is likely to apply to *tk64*.

*Bajan* contains a splice site mutation which creates a premature stop codon in all of its transcripts upstream of the catalytic site. Injection of morpholinos directed against the translation start site of *bajan*, blocking any potential residual translation, was shown to be early lethal [17]. Together, these results [17] indicate that some residual zebrafish ChATa activity persists in *bajan*. This activity could be either due to low-level alternative splicing of *chata*, or to upregulation of a compensatory pathway (perhaps involving the paralogous gene, *chatb* [23] via nonsense-mediated mRNA decay [24]). Both mechanisms would work only in *bajan* mutants, but not in the morphants.

In contrast to *bajan*, we attribute the residual motility found in the *tk64* allele to an altered, rather than a truncated ChAT protein, preventing any effect of nonsense-mediated decay. The mutated residue serine S102 is localized next to the catalytic site as inferred from the crystal structure of human ChAT (Fig 5C). In comparison to the published protein crystal structure (PDB ID 2fy2), the S97R change replaces the buried polar uncharged serine residue by a charged arginine and creates new H-bonds with the residues glutamine 309 and serine 307, both residing in the catalytic domain, which probably effect catalytic activity via incorrect protein folding and altered physiochemical properties. Alternatively, a direct involvement of S97 in catalysis cannot be ruled out [25–27]. Moreover, a motif containing S97 has been identified as a potential phosphorylation site of ChAT [28].

In humans, so far a total of 32 missense or nonsense mutations have been reported in ChAT [4,6,13,29–35], out of which 15 mutant alleles (all missense mutations), causing CMS-EA, affect the catalytic domain of the protein [4,6,13,30–33]. Measurement of ChAT enzyme activity and catalytic efficiency in studies by Arredondo et al. [7] and Ohno et al. [2] shows that overall catalytic efficiency considerably decreases compared to that of wild-type ChAT resulting in a range of activity levels. CMS patients carrying a V18M mutation (distal to active site of ChAT) exhibit fourfold reduction of *in vitro* catalytic efficiency due to decreased enzyme affinity for acetyl-CoA [13]. It was also reported that this mutation resulted in a decreased steady state ChAT protein level in BOSC23 cells.

Morey et al 2016 [36] elucidated ChAT function and cellular stability by assessing the effect of the highly conserved proline rich motif (14)PKLPVPP(20) surrounding the mutated valine residue. Mutation of prolines in this motif (P17A/P19A) shows a dramatic decrease in both ChAT half-life and steady state protein levels in neural cells. This disruption of the proline rich motif was shown to enhance ubiquitination and proteosomal degradation of the mutated ChAT protein. Furthermore, molecular chaperones like HSP70/HSP90 were shown to interact with ChAT, regulating protein stability by compromising protein folding and promoting proteosomal degradation of ubiquinated mutated ChAT proteins (V18M, A513T) [37]. Our mutation on the other hand is in the catalytic domain and is expected to affect the protein conformation. An effect of the mutation on enhancing ubiquitin-proteosome mediated degradation is less likely since this region is neither proline nor lysine rich as target motifs for ubiquitination commonly are [38,39].

We conclude that our mutant, *chata*^*tk64*^ is the closest zebrafish model created so far for human presynaptic CMS caused by missense mutations in ChAT. This model provides information on a critical amino acid which can hamper the activity of choline acetyltransferase enzyme resulting in impaired motility, if mutated. Therefore, it can help to better understand the etiology of myasthenic syndromes.

## Materials and methods

### Ethics statement

All zebrafish husbandry work was performed in accordance with the German Animal Welfare Act and was approved by the Regierungspräsidium Karlsruhe, Germany. A permit for experimental procedures or euthanasia was not required since no such procedures were carried out later than 5 days post fertilization.

### Fish stocks

Fish were obtained from frozen sperm stored at the European Zebrafish Resource Center (EZRC). The fish were outcrossed against the AB wild-type line and raised as previously described by Westerfield (1993) [40].

### Immunohistochemistry

Immunohistochemistry was performed by standard methods using anti-α-actinin (Sigma); anti - α -sarcoglycan (Novocastra); F59, which is specific to slow-muscle myosin isoforms (slow-MHC, DSHB); anti-titin (T11; Sigma), which marks an epitope in the I-band region of titin [41]; and monoclonal anti-acetylated tubulin, clone 6-11B-1 (Sigma). Alexa Fluor-conjugated phalloidin or bungarotoxin (Invitrogen) was applied after the secondary antibodies. Optical sections were taken with a Leica TCS4D confocal microscope.

### Library preparation

Whole genome DNA sequencing was performed with a HiSeq 1500 machine (Illumina) and a compatible indexed library was generated as follows. The concentration of genomic DNA (ng/μl) was quantified by the fluorometric method using Qubit (Thermo Fisher Scientific Inc.). The amount equivalent to 1 μg DNA was processed with the S220 Focused-ultrasonicator (Covaris Ltd, Brighton UK) in a glass vial (microTUBE AFA Fiber Pre-Slit Snap-Cap 6x16mm, Covaris Ltd) to generate 350 bp DNA fragments which were used for indexed DNA library preparation using the TruSeq Nano DNA LT Library Preparation Kit following the protocol from Illumina. Briefly, after the sonication the overhangs of DNA fragments were converted to blunt ends and a single adenine nucleotide was added to the 3’ ends for optimal adapter ligation. Purified adapter-ligated DNA fragments were amplified by PCR (polymerase chain reaction; 12–14 cycles) to enrich genomic DNA fragments. Quantity and quality of DNA library was assessed with an Agilent 2100 Bioanalyzer using either a High Sensitivity DNA chip or DNA 1000 chip. The DNA library (if necessary multiplexed) at 10 pM was used for cluster generation on a high-throughput sequencing flow cell using the cBot system (TruSeq PE Cluster Kit v3), aiming at a cluster density of 750–850 k/mm2. Paired-end 50-bp sequencing was performed with the HiSeq 1500 using TruSeq SBS Kit v3 reagents.

### Mutation mapping

#### Quality assessment, pre-processing and alignment

Raw reads from both mutant and wild-type samples were assessed for quality using FASTQC v0.11.4 [42]. Paired-end reads were aligned to the zebrafish reference genome GRCz10 assembly using the Burrows-Wheeler aligner (BWA v 0.7.12-r1039) commands “-aln” and “sampe” with parameters “-o 1 -n 0.01 -d 12 -e 12 -q 20” [43,44]. For better mapping results these optimum BWA parameters were used to yield higher mapping percentage [44]. Reads were also trimmed in the alignment process to remove the low quality bases which assisted in high quality variant calling [45]. The resulting BAM file was sorted by coordinates and duplicate reads were marked using picard v1.40 [46]. Base quality score recalibration, indel re-alignment, recalibration were performed using GATK v3.7-0-gcfedb67 [47,48] to output analysis ready recalibrated BAM files of mutant and wild-type siblings. After pre-processing steps a mapping coverage of approximately 9x (320,805,334 reads) for the mutant and 8x (277,089,644 reads) for the wild-type sample was attained.

#### Variant calling, linkage mapping, variant ranking and annotation

Our linkage mapping strategy will be described in detail elsewhere (manuscript in preparation). In brief, variant calling (with genotyping) was performed on mutant and wild-type BAM files together, on all chromosomes in parallel, using GATK-HaplotypeCaller [48] (with the “—stand_call_conf 10.0” option) to yield two sample VCF file for each chromosome. These were analysed in two stages: In the first stage, GATK best practices recommendations for hard filtering parameters were used to remove low quality SNPs and indels [48,49]. Additionally, variants with genotype quality (GQ) less than 5 and multi-allelic variants were removed. To map the region encompassing the SNP markers linked to the mutation–which are homozygous in mutants and heterozygous or homozygous for the reference sequence in the wild-type pool–a custom algorithm implemented in Python 2.7 and R (version 3.2.3) was used (manuscript in preparation). The chromosome with the highest homozygosity (Fig 2A) was selected and the linked region was determined based on the maxima of the LOESS fit (Fig 2B). In the second stage we used VariantMetaCaller (VMC) to obtain high quality variants [50]. VMC incorporates annotations generated by different variant callers in a single score metric. The variant callers were samtools “mpileup” (with the -C50 option) [51], platypus v0.8.1 [52] (using default parameters), and freebayes v0.9.9.2 (“-q20” option, only for SNPs with no multi alleles) [53]. Before applying VMC, additional annotations available from the variant callers such as Shannon entropy and distance from next variant were also added to all the VCF files using vcflib [54] and bcftools v1.3 [55]. From the final output VCF file generated by VMC, variants which were homozygous (1/1) in the mutant sample and heterozygous or homozygous for the reference sequence (0/1 or 0/0) in the sibling sample were extracted and annotated using the Ensembl (version 87) variant effect predictor (VEP) [19]. Variants were ranked according to their impact rating as predicted by VEP [19]. Variants which were found to already exist in the dbSNP database were filtered out. Only variants with high and moderate impact rating were retained.

### ChAT protein structure modelling and mutation analysis

The protein structure of the human homolog ChAT PDB ID 2fy2 [11] was used to model the structural effect of the amino acid change using UCSF chimera v1.11.2 [56]. Structure editing (S97R) was done using the rotamer with the highest probability [57]. Structure minimization was performed after structure analysis (Find clashes/contacts command) with default parameters. Hydrogen bonds were also measured using default parameters with the “FindHBond” command in both wild-type and mutant structure for serine and arginine respectively.

## Supporting information

S1 Video Touch response of wild-type embryo at 48 hpf. Wild-type embryos escape in response to touch.
(AVI)

S2 Video Touch response of mutant embryo at 48 hpf. Mutant embryos couldn’t escape, only twitch in response to touch.
(AVI)
